# Progression of aortic regurgitation following transcatheter closure of intracristal ventricular septal defects in children: a mid- to long-term follow-up study

**DOI:** 10.3389/fcvm.2023.1190013

**Published:** 2023-05-03

**Authors:** Qiuman Li, Xu Zhang, Yukai Xu, Lingmei Zhou, Junjie Li, Zhiwei Zhang

**Affiliations:** ^1^ Department of Pediatric Cardiology, Heart Center, Guangzhou Women and Children's Medical Center, Guangzhou, China; ^2^ Department of Pediatric Cardiology, Guangdong Cardiovascular Institute, Guangdong Provincial People's Hospital (Guangdong Academy of Medical Sciences), Southern Medical University, Guangzhou, China; ^3^ School of Medicine, South China University of Technology, Guangzhou, China

**Keywords:** intracristal ventricular septal defect, aortic regurgitation, transcatheter closure, risk factor, follow-up

## Abstract

**Background:**

Early surgical closure is warranted to prevent aortic valve lesion and aortic regurgitation (AR) in intracristal ventricular septal defects (icVSDs). Experiences for transcatheter device closure of icVSDs are still limited. Our objectives are to investigate AR progression following transcatheter closure of icVSDs in children and to explore the risk factors for AR progression.

**Methods and results:**

From January 2007 to December 2017, 50 children with icVSD who had successfully undergone transcatheter closure were enrolled. With 4.0 (interquartile range: 3.0–6.2) years of follow-up, AR progression was observed in 20% (10/50) of patients after icVSD occlusion, among which 16% (8/50) remained in mild level and 4% (2/50) evolved to moderate. None progressed to severe AR. Freedom from AR progression was 84.0%, 79.5%, and 79.5% at 1, 5, and 10 years of follow-up. A multivariate Cox proportional-hazards model revealed that x-ray exposure time [hazard ratio (HR): 1.11, 95% confidence interval (CI): 1.04–1.18, *P* = 0.001] and the ratio of pulmonary to systemic blood flows (HR: 3.38, 95% CI: 1.11–10.29, *P* = 0.032) were independent predictors for AR progression.

**Conclusions:**

Our study suggested that transcatheter closure of icVSD in children is safe and feasible in mid- to long-term follow-up. No serious AR progression occurred after icVSD device closure. Greater left-to-right shunting and longer x-ray exposure time were both risk factors for AR progression.

## Introduction

Infundibular ventricular septal defect (VSD) is historically named as subarterial, supracristal, subpulmonic, or conal VSD (1–5). Due to the proximity to the semilunar valves, infundibular VSD carries a high risk of aortic valve prolapse (AVP) with subsequent progression of aortic regurgitation (AR) (5–7). Therefore, early closure is warranted in these patients (5, 6).

Percutaneous transcatheter device closure has increasingly been accepted as an alternative to surgical repair in selected cases of VSD (8), especially muscular or perimembranous ones. Whether the transcatheter procedure plays a role in infundibular VSDs, however, remains poorly investigated. On the basis of the classification system of VSD from ICD-11 (4), the alleged infundibular VSD should be further divided into two subcategories: the doubly committed juxta-arterial VSD, and the muscular outlet VSD. The former is contraindicated for transcatheter closure in the absence of a superior rim to reliably support the occluder (9). The latter might have a chance because the muscular rim exists beneath the pulmonary valve. In recent years, several investigators have explored this feasibility (10–14). In their works, the defect was referred to as “intracristal.”

To this day, it is technically feasible to close intracristal VSDs (icVSDs) with various devices, with reported success rates of 79%–94% (10–14). Perioperative major complications are rare, with encouraging short-term follow-up results in adults. The conduction complications are less frequent. However, the influence of the device on the aortic valve is still raising concern. There is a high risk for AVP and AR before operation in outlet VSD. Recent research revealed that AR may progress even after open-thoracic surgery (7). Will that progression exist after device closure? What are the risk factors for that? Since the rim is deficient to the aortic valve, the device might keep in touch with the valve in most cases. Will this “intimate contact” bring long-term adverse outcome such as valve perforation? In this study, we reported our experiences and results of transcatheter closure of icVSDs in children in a mid- to long-term follow-up, focusing mainly on the fate of aortic valve and the corresponding risk factors.

## Methods

### Patient selection

From January 2007 to December 2017, 50 children (<18 years) with icVSD that were successfully closed percutaneously were enrolled in this study. Significant left-to-right shunt was the most important indications for interventional occlusion. Most of the children met at least one of the following criteria: (a) failure to thrive; (b) recurrent respiratory infections; (c) cardiomegaly on chest x-ray; (d) enlargement of left atrial and/or left ventricle on transthoracic echocardiography (TTE); (e) AVP already existed. Exclusion criteria were as follows: (a) combined with other congenital heart defects if simultaneous surgery were needed; (b) severe pulmonary artery hypertension or right to left shunt; (c) congenital aortic valve malformations, such as bicuspid aortic valve; (d) moderate-to-severe AR; (e) sepsis; (f) body weight <10 kg, and (g) contraindication to antiplatelet therapy. The study was approved by the Research Ethics Committee of the Guangdong General Hospital (No. GDREC2020213H).

### Definitions

**Figure 1:**
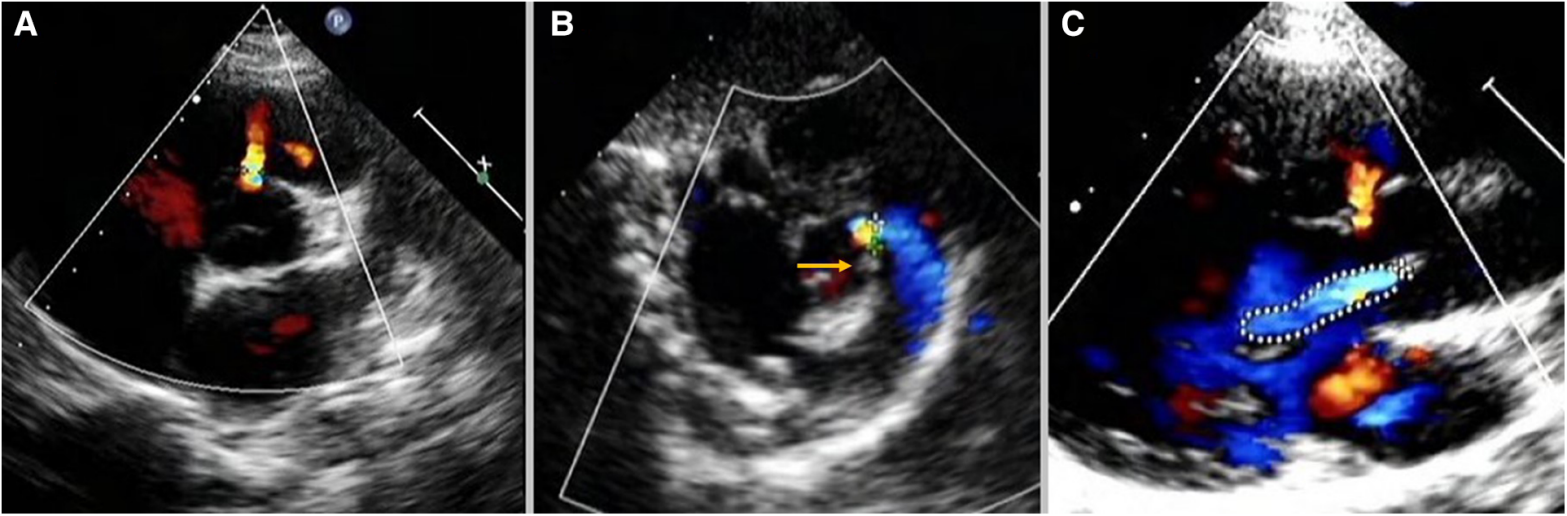
Intracristal VSD was diagnosed on TTE: (**A**) in parasternal short-axis sections, the defect was demonstrated at approximately 12-o’clock with the aortic valve seen face on. (**B**) The yellow arrow shows the presence of subpulmonary infundibulum precluded the diagnosis of doubly committed juxta-arterial VSD. (**C**) The defect was adjected to the right aortic valve leading to AVP and AR. TTE, transthoracic echocardiography; VSD, ventricular septal defect; AVP, aortic valve prolapse; AR, aortic regurgitation.

IcVSD was preoperatively diagnosed on TTE (Figure 1). In parasternal short-axis sections, with the aortic valve seen face on, the defect was demonstrated at approximately 12 o’clock, indicating its opening into the right ventricular outlet region (Figure 1A). Doubly committed juxta-arterial and perimembranous VSDs were both excluded (Figure 1B). The aortic valve was thoroughly evaluated for AVP and AR (Figure 1C). AR severity assessment was previously described in detail by the ratio of maximal AR jet width within 1 cm of the aortic valve compare with the left ventricle outflow tract diameter (15, 16). According to the recommendations, a <25% ratio represents mild AR; 25%–64% represents moderate AR; ≥65% represents severe AR. AR progression was defined as the progression of valve regurgitation from one grade to the more advanced grade.

The distance between VSD and right coronary cusp was determined by both TTE and angiography. A deficient rim to the aortic valve was defined as the distance <2 mm, and a sufficient rim was ≥2 mm.

### Devices

Multiple types of devices were used in this study. They were all modified double-disk occluders made from nitinol wire. Both symmetric and eccentric shape VSD occluders were manufactured by LifeTech Scientific, Shenzhen, China, or by Starway Medical, Beijing, China. Amplatzer Duct Occluder (ADO) I or II devices (St. Jude Medical, St. Paul, MN, United States) were also used in selected cases.

### Procedure

All patients underwent routine left and right cardiac catheterization. TTE was performed preoperatively. Left ventriculography and aortic root angiography were both performed to confirm the relationship between the aortic valve and icVSD. Thereafter, an appropriate occluder was selected according to the size and morphology of icVSD, sufficiency or deficiency of the superior rim to the aortic valve, as well as the presence or absence of AVP. Typically, the original selected size was 2–3 mm larger than the defect. If the superior rim is sufficient, the symmetric occluder was selected. Otherwise, the eccentric occluder was preferred. In very small defects, ADO II might also be an alternative. Before the release of the occluder, left ventriculography and aortic root angiography, as well as TTE, were repeated to confirm the appropriate position of the device without influence on the morphology and function of the aortic valve. TTE was also performed to monitor the whole procedure.

### Follow-up

All patients were carefully followed after occlusion. Standard 12-lead electrocardiography and TTE were regularly followed up at 1 day, 1 month, 3 months, 6 months, and 12 months after the procedure, and every 1–2 years thereafter. All patients received oral aspirin at 3–5 mg/kg per day for 6 months after the procedure.

### Data collection

Clinical data were collected from chart review. The first author was responsible for data acquisition (QL), including patient demographics, echocardiographic parameters, transcatheter procedural data, and other associated complications. Echocardiographic parameters included VSD size, left ventricular end-diastolic diameter (LVEDD), left ventricular ejection fraction (LVEF), the presence or absence of AVP, AR, residual shunt, and AR severity. Transcatheter procedural data included mean pulmonary arterial pressure (mPAH), pulmonary to systemic flow ratio (*Q*_p_/*Q*_s_), indexed pulmonary vascular resistance (PVRI), x-ray exposure time, device sizes, and device types. Device types were based on their geometric shape: (1) eccentric type: VSD occluder; (2) other types included symmetric-type VSD occluder and ADO I/II.

### Statistical analysis

We expressed categorical variables as percentages while continuous variables as median (interquartile range, IQR) or mean (standard deviation, SD). Comparison of categorical variables was analyzed using the Chi-square test or Fisher's exact test. Two-sided unpaired Student’s *t*-test or Wilcoxon rank sum test was performed for comparison of continuous variables appropriately. The Kaplan–Meier method was performed to draw event-free survival curves, while those survival curves were compared with the log-rank test. Predictors of AR progression was identified with Cox proportional-hazards model and expressed as hazard ratio (HR) and 95% confidence interval (CI). *P* values <0.05 were defined as statistically significant. All statistical analyses were performed with SPSS 21.0 (IBM Corp., Armonk, NY, United States).

## Results

### Baseline characteristics

The median follow-up durations of all icVSD children were 4.0 (IQR: 3.0–6.2) years. The follow-up rate is 98.0%, 84.0%, 84.6%, and 81.3% at 1-year, 3-year, 5-year, and 7-year periods (Table 1).

**Table 1:** The follow-up rate of icVSD children after VSD occlusion.

	1-year	3-year	5-year	7-year
Follow-up rate, %	98.0 (49/50)	84.0 (42/50)	84.6 (22/26)	81.3 (13/16)

VSD, ventricular septal defect.

The baseline characteristics are demonstrated in Table 2. Mean age at closure was 4.35 (IQR: 2.98–6.40) years. Mean VSD size was 3.99 (SD: 0.99) mm. Mean *Q*_p_/*Q*_s_ was 1.57 (SD: 0.46). AVP was recorded in 45 (90%) patients, among whom 25 (55.6%) had both right coronary cusp prolapse (RCP) and noncoronary cusp prolapse (NCP), 14 (31.1%) had RCP only, and 6 (13.3%) had NCP only. 8.9% (4/45) of icVSD with AVP developed mild AR before occlusion. For devices used, 54.0% (27/50) were eccentric-type occluders, 36.0% (18/50) were symmetrical type, and other devices used were ADO I (4/50) and ADO II (1/50).

**Table 2:** Baseline characteristics of entire cohort before icVSD occlusion.

	*N* = 50
Sex, male	31 (62%)
Age (years)	4.35 (2.98–6.40)
Age <3 years	12 (24%)
Weight (kg)	16.00 (13.38–22.75)
VSD size by TTE (mm)	3.99 (0.99)
LVDD (mm)	34.61 (4.19)
LVEF (%)	71.88 (5.86)
*Q*_p_/*Q*_s_	1.57 (0.46)
mPAH (mmHg)	14.96 (3.69)
PARI (wood × m^2^)	1.99 (0.83)
Rim of VSD to RCP	
Deficient (<2 mm)	43 (86%)
Sufficient (≥2 mm)	7 (14%)
AVP	45 (90%)
Both RCP and NCP	25 (55.6%)
RCP only	14 (31.1%)
NCP only	6 (13.3%)
AR severity	
Mild	4 (8%)
Moderate to Severe	0
Occluder	
Eccentric type	27 (54%)
Symmetrical type	18 (36%)
PDA occluder	5 (10%)
Total catheterization time (min)	78.70 (29.22)
x-ray exposure time (min)	14.28 (7.39)

VSD, ventricular septal defect; TTE, transthoracic echocardiography; LVDD, left ventricular end-diastolic diameter; LVEF, left ventricular ejection fraction; *Q*_p_/*Q*_s_, pulmonary/systemic flow ratio; mPAH, mean pulmonary artery pressure; PARI, pulmonary artery resistant index; AVP, aorta valve prolapse; RCP, right coronary cusp prolapse; NCP, noncoronary cusp prolapse; AR, aortic regurgitation; IQR, interquartile range; PDA, patent ductus arteriosus.

Data are expressed as number (percentages), median (IQR), or mean (SD).

### AR progression

**Figure 2:**
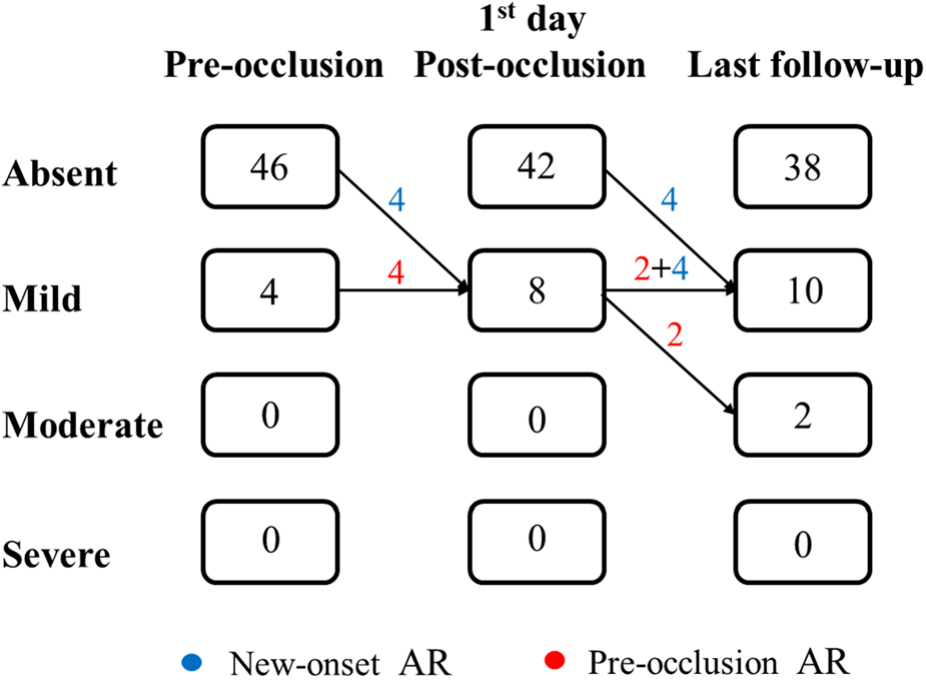
Progression of AR degrees during follow-up. AR, aortic regurgitation.

**Figure 3:**
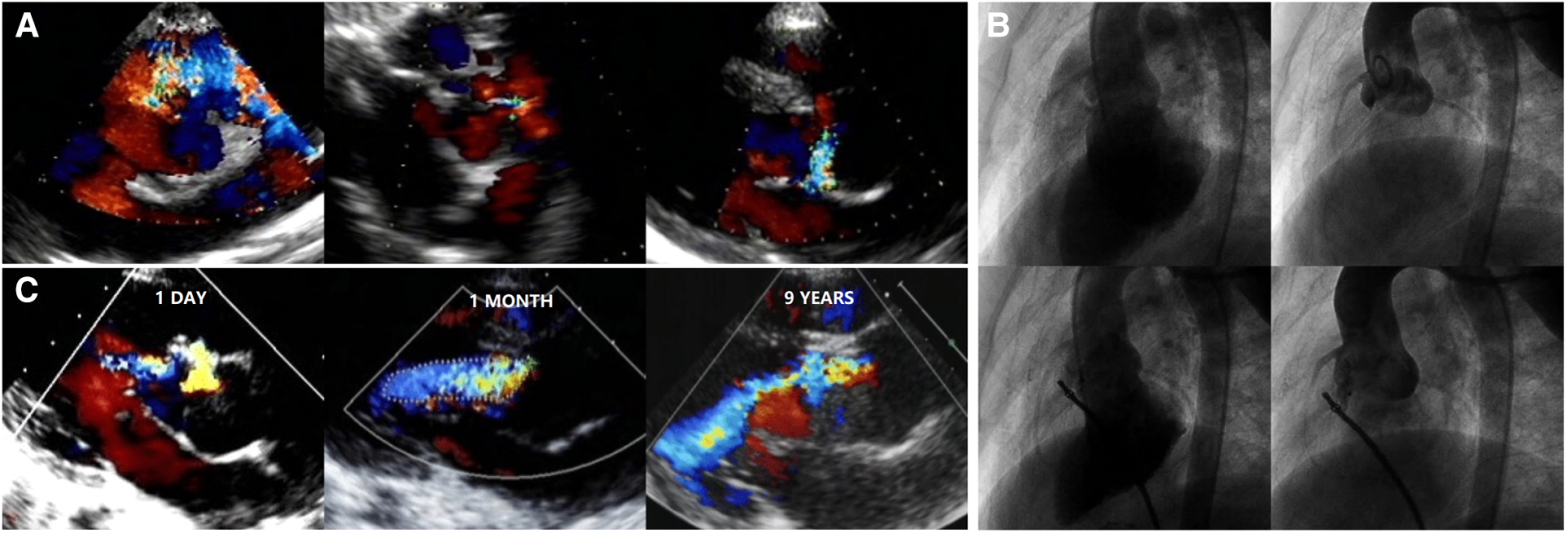
TTE and angiography in an icVSD case using a symmetrical VSD occluder: (**A**) features of the icVSD on TTE. (**B**) Features of the icVSD on angiography and a symmetric VSD occluder was used to close it with no obvious AR progression instantly. (**C**) AR remained mild at 1 day and progressed to moderate at 1 month and 9 years after the VSD occlusion. TTE, transthoracic echocardiography; VSD, ventricular septal defect; AR, aortic regurgitation.

**Figure 4:**
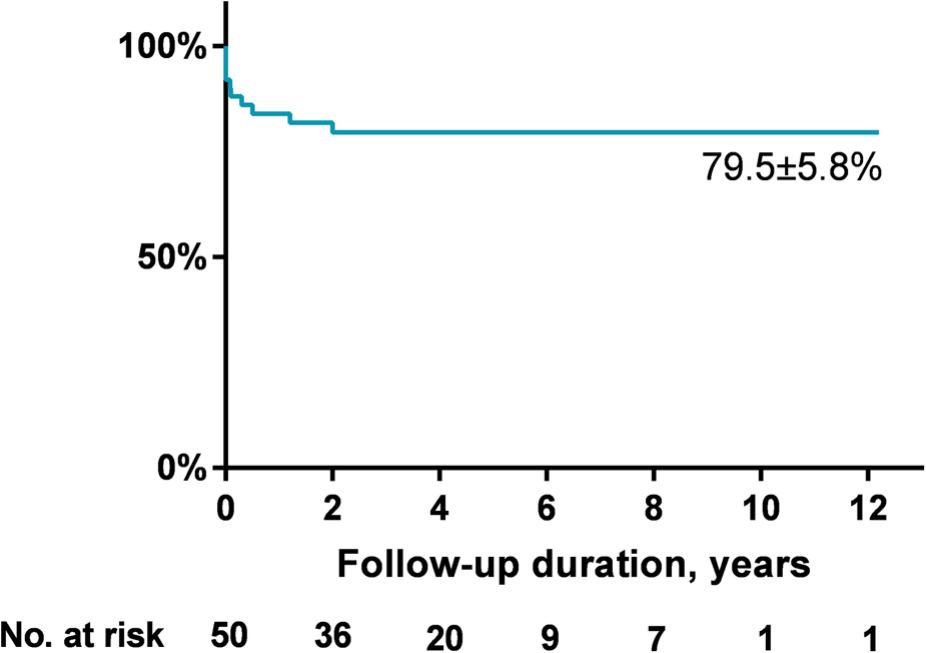
Freedom from AR progression of the entire cohort. AR, aortic regurgitation.

AR progression was demonstrated in Figure 2. AR progression was observed in 20% (10/50) of the patients. New-onset mild AR was recorded in 16% (8/50) of the patients, among which 8% (4/50) were detected at the first day after occlusion and 8% (4/50) occurred later. There are four patients with mild AR before occlusion, two of which progressed to moderate while the other two remained in mild level. One moderate AR was detected at 1 month and another at 6 months by TTE after VSD closure. For the two early cases that developed moderate AR, both used symmetrical occluders (Figure 3). For the entire group, freedom from AR progression was 84.0%, 79.5%, and 79.5% at 1, 5 and 10 years of follow-up (Figure 4). AR progression terminated 3 years after VSD closure and remained stable thereafter. No cases developed severe AR, and no valvuloplasty or valve replacement were needed.

### Predictors of AR progression

**Figure 5:**
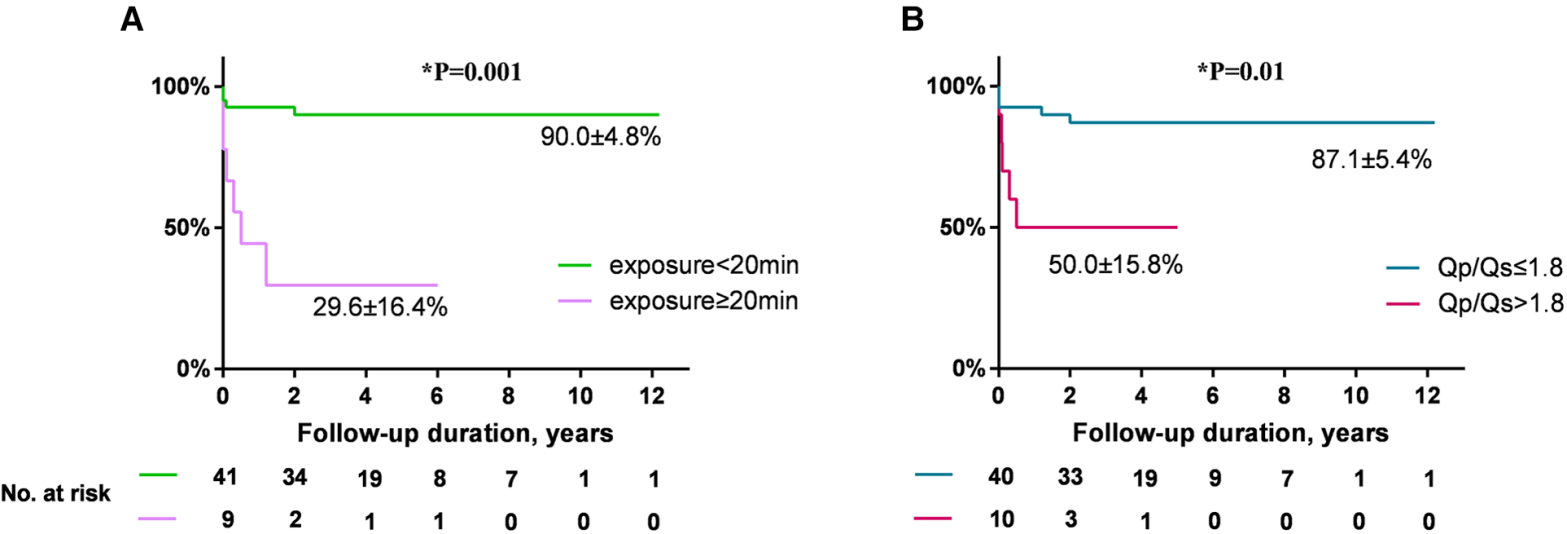
(**A**) Comparison of freedom from AR progression by x-ray exposure time (exposure ≥20 min vs. <20 min, HR: 9.78, 95% CI: 2.68–35.71). (**B**) Comparison of freedom from AR progression by *Q*_p_/*Q*_s_ (*Q*_p_/*Q*_s_ > 1.8 vs. ≤1.8, HR: 5.20, 95% CI: 1.48–18.27). AR, aortic regurgitation; HR, hazard ratio; CI, confidence interval.

Both univariate and multivariate analysis of AR progression is demonstrated in Table 3. Univariate analysis revealed that *Q*_p_/*Q*_s_, the size of selected occluder, and x-ray exposure time were significantly associated with AR progression. Age or weight at presentation, AR before occlusion, VSD size by TEE, and occluder type (eccentric vs. other types) were not predictive of AR progression. A multivariate Cox proportional-hazards model revealed that x-ray exposure time (HR: 1.11, 95% CI: 1.04–1.18, *P* = 0.001) and *Q*_p_/*Q*_s_ (HR: 3.38, 95% CI: 1.11–10.29, *P* = 0.032) were independent risk predictors for AR progression. Patients with exposure time longer than 20 min had almost 10-fold risk of AR progression compared with those less than 20 min (HR: 9.78, 95% CI: 2.68–35.71, *P* = 0.001) (Figure 5A). Patients who had *Q*_p_/*Q*_s_ > 1.8 had five-fold risk of AR progression compared with those who had *Q*_p_/*Q*_s_ ≤ 1.8 (HR: 5.20, 95% CI: 1.48–18.27, *P* = 0.01) (Figure 5B).

**Table 3:** Predictors of AR progression.

	Univariate analysis		Multivariate analysis*	
	HR (95% CI)	*P* value	HR (95% CI)	*P* value
Age, years	1.14 (0.97–1.34)	0.12		
Male vs. female	1.60 (0.41–6.20)	0.50		
Weight, kg	1.05 (1.00–1.10)	0.063		
AR vs. no AR pre-occlusion	3.13 (0.66–14.80)	0.15		
Rim of VSD to RCP (deficient vs. sufficient)	0.038 (0.00–62.64)	0.39		
x-ray exposure time, min	1.13 (1.06–1.20)	<0.001	1.11 (1.04–1.18)	0.001
VSD size by TTE, mm	1.57 (0.82–3.03)	0.18		
*Q*_p_/*Q*_s_	3.38 (1.51–7.59)	0.003	3.38 (1.11–10.29)	0.032
Occluder size, mm	1.69 (1.13–2.51)	0.010		
Occluder types (eccentric vs. other types)	1.30 (0.37–4.62)	0.68		

AR, aortic regurgitation; VSD, ventricular septal defect; RCP, right coronary cusp prolapse; TTE, transthoracic echocardiography; *Q*_p_/*Q*_s_, pulmonary/systemic flow ratio; HR, hazard ratio; CI, confidence interval.

### Other complications

Residual shunt was observed in six cases immediately after occlusion but all disappeared during follow-up. Four patients experienced transient arrhythmias (including one third degree atrioventricular block, one nonspecific QRS interval prolongation, one junctional escape rhythm, and one nonspecific ST-T change) intraoperatively or postoperatively, which resolved spontaneously and did not recur. Two patients had new-onset incomplete right bundle branch block after procedure. Neither death nor surgical operation occurred due to complications following transcatheter intervention.

## Discussion

Aortic valve lesions are common in the natural history of outlet VSDs (5–7, 17–20). Previous studies identified that the “Venturi effect” is the predominant cause for that (21). Due to the proximity of the aortic valve leaflets to the defect, a pressure gradient was formed from the valve to the defect, pulling the aortic valve cusps toward the shunting. Previous reports support this concept, demonstrating that AVP and AR are five times more common in infundibular VSDs than in perimembranous VSDs (21–23).

In this study, we thoroughly detected the presence of aortic valve lesions and the potential AR progression after transcatheter closure of intracristal VSDs in children in mid- to long-term follow-up. In our series, 90% of patients had AVP and four had mild AR before VSD closure, which is consistent with the notion that aortic valve lesion is common in the natural history of outlet VSDs. Several studies in pediatric cohorts with outlet VSDs suggested that the risk of evolving aortic insufficiency increases during childhood, and the peak age of the emergence of AVP and AR was between 5 and 10 years, respectively (17–20). Therefore, early closure is warranted in such patients.

Traditionally, open-thoracic surgery is the exclusive option for outlet VSD patients (21, 24). With the advent of new devices, percutaneous transcatheter closure of muscular and perimembranous VSD became an alternative in selected cases in many tertiary centers. However, attempts in outlet VSDs are still rare, for the concern of several complications. First, the superior rim to the semilunar valves is deficient, which could increase the risk of device embolization. Moreover, the aortic valve might suffer from deformation by device extrusion, contributing to aortic insufficiency. In the preceding decade, several groups of researchers recognized a subset of the so-called icVSD (10–14). The icVSD is actually a subtype of outlet muscular VSD in infundibular VSD, the characteristics of which are the presence of residual subpulmonary infundibulum (although almost all of them are hypoplastic), which clearly differentiates it from doubly committed juxta-arterial VSD in which the subpulmonary infundibulum is totally absent, leading to the diagnostic fibrous continuity between the leaflets of the semilunar valves (9). The residual infundibulum could theoretically provide support for stabilization of the device. Therefore, there is a possibility to perform transcatheter closure in these patients.

Previous researchers made initial transcatheter attempts in icVSDs, with acceptable success rates and in early-to-mid-term follow-up results (10–14). Device embolization was uncommon in these series. The aortic valve, despite the use of eccentric or other specialized devices, demonstrated high rates of insufficiency. Gu et al. employed perimembranous occluders (86% are eccentric type) for the closure of icVSDs and found that 10.2% (5/49) of patients experienced new-onset trivial-to-mild aortic valve regurgitation immediately after device closure, and all AR remained stable during an average of 2.3 years of follow-up (10). Another study from the same group reported the application of a specialized zero eccentricity occluder (no superior margin of the left disc extending toward the aortic valve) for the purpose of avoiding aortic valve lesion, which found that 13.2% (5/38) of patients developed new-onset AR after VSD closure immediately and all AR remained stable during 1.4 (range: 0.3–2) years of follow-up (12). Other studies from different centers achieved similar results: a range of 5.4%–15.4% of icVSDs experienced new-onset or aggravated AR after device closure or leading to aborted procedure (11, 13, 14). The new-onset or aggravated AR should largely be device-related. But most of them, once the technical success was achieved, seemed to be mild and remained stable during follow-up.

Up to date, however, data are limited with regard to the mid- to long-term results of aortic valves, and most cases included in the above studies were adults (e.g., the mean ages at intervention in the studies by Gu et al. and Chen et al. were 18.5 and 25.3 years, respectively), in a relatively short follow-up period of time. Experiences in pediatric patients are scarce. Our study was the first report in children with transcatheter intervention, mostly at preschool ages. This time of point is before the peak age of the emergence of aortic valve lesions in natural history (17–20), which provides a good opportunity to observe whether transcatheter intervention could improve the aortic valve outcome or not. In practice, RV outlet space in children will be enlarged along with physical development, and the device might subsequently become out of contact with the aortic valve after outlet enlargement. So, device-related AR in children might theoretically have a tendency of spontaneous remission. Unfortunately, we did not observe this hypothetical improvement. In our series, 20% of total cases (10/50) exhibited an AR progression after device closure. Among them, four cases with immediate new-onset AR were detected at the first day after transcatheter closure, which were all mild and probably device-related. Another four cases experienced late-onset mild AR. In the four previously existed mild AR before closure, two progressed to moderate level during follow-up. The reasons for these six late progressions were unclear. Close contact of aortic valve leaflets to the devices increased the risk of valve erosion, but this possibility was not evidenced during imaging. Another possibility is the natural progression after initiation of aortic valve lesion. This is consistent with previous reports that a subset of patients with outlet VSD would experience progression of AR after surgical repair (6, 7, 25–27), and it is accepted worldwide that early intervention could prevent the worsening of AR. Our series demonstrated similar results. Since surgery is the well-recognized option to manage VSD patients with prominent AR, all cases included in our series were VSDs with no AR or mild AR preoperatively. After transcatheter intervention, freedom from AR progression was seen in 84.0%, 79.5%, and 79.5% of patients, at 1, 5, and 10 years of follow-up. Only two cases progressed to moderate level, and no patient developed severe AR without any need for valvuloplasty or valve replacement. Based on our results, we believe that transcatheter closure of icVSD is safe and feasible in children at preschool age.

Up to now, no research study had reported any predictors of post-occlusion AR progression in icVSD. We first discovered that three factors are associated with AR progression after device closure: bigger occluder size used for VSD closure, *Q*_p_/*Q*_s_, and longer x-ray exposure time. After that, we further identified that both higher *Q*_p_/*Q*_s_ and longer x-ray exposure time are independent risk factors for AR progression. One explanation for these results is that the prolapsed right coronary cusp usually covers the defect, resulting in an increased measurement error, which not only affects the selection of device but also increases the procedural difficulty, especially in those with larger VSDs. On the other hand, the more difficulty we faced in the procedure, the more time of exposure was needed under fluoroscopy. Also, prolonged manipulation of catheters and guide wires around the aortic valve might increase the risk of damage to the leaflets, which could contribute to postoperative AR progression. Our results suggested that no more than 20 min of exposure time may decrease AR progression. Skilled manipulation techniques could not only reduce the x-ray radiation exposure but also reduce the risk of AR progression. When *Q*_p_/*Q*_s_ > 1.8, transcatheter intervention should be performed with great caution. If guidewire tracking for intervention needs to be established several times or larger occluders need to be sequentially attempted, transcatheter intervention should be aborted and surgical thoracotomy may be considered.

Two cases in our series developed moderate AR during follow-up. They both used symmetrical type VSD occluders. This reminded us that the symmetrical occluder should be avoided in icVSD. Moreover, careful evaluation of the relationship between the occluder and aortic valve, as well as their positions and shapes, should be repeated by TTE and angiography before occluder release. We noticed that the eccentric occluder led to less structural deformity to aortic valve, although the rate of AR progression was not decreased in this group. Based on these experiences, we believe that an eccentric occluder might be more suitable for icVSD closure, which is consistent with experiences from the report of Chen et al. (12). Recently, Tang et al. reported using ADO II to close small doubly committed subarterial VSDs (1.5–3.5 mm) (28). In their short-term study (1–45 months), only 1/24 developed new-onset mild AR, which was similar to our recent report (1/13) (28, 29). Therefore, ADO II may be one of the options for small icVSD because its material is softer than other occluders, which may preclude sustained damage to the aortic valve. However, this opinion still needs longer-term follow-up for more evidence.

In our study, neither significant residual shunting nor severe arrhythmias were demonstrated. This is comprehensible because icVSD is anatomically in the region of muscular outlet and relatively far from the bundle of His and bundle branches. So, device-related arrhythmias were not common in transcatheter icVSD closure and could be a minor problem of concern. This is also consistent with other series that included adults (10–14).

### Limitations

Our study has limitations. First, this is a single-center retrospective study with a relatively small sample in pediatric population. Comparison with surgical intervention was not performed. So, we could not know if transcatheter device closure could replace surgical repair in selected cases. Second, occluder selection was based on previous experiences extrapolated from perimembranous VSDs, and no uniform criteria were employed in our study. So, comparison between differential types of occluders was not accurate. We could not reach a conclusion as to which type of occluder is better. Further multicentered randomized controlled studies are needed to solve these questions.

## Conclusions

Our study demonstrated that the incidence of AR progression after percutaneous transcatheter device closure of icVSD in preschool pediatric patients is no higher than that in previously reported adult cohorts. The majority of AR remained unaltered at mild level and only few cases developed to moderate. No severe AR occurred and neither valvuloplasty nor valve replacement were needed during mid- to long-term follow-up. We also identified that greater left-to-right shunting and longer x-ray exposure time were both independent risk factors for post-occlusion AR progression. Our results suggested that transcatheter closure of icVSD in preschool children is safe and feasible in mid- to long-term follow-up. We recommend performing this procedure in experienced tertiary cardiac centers.

## Data Availability

The original contributions presented in the study are included in the article, further inquiries can be directed to the corresponding authors.
